# Neurodegenerative diseases in a dish: the promise of iPSC technology in disease modeling and therapeutic discovery

**DOI:** 10.1007/s10072-014-1989-9

**Published:** 2014-10-30

**Authors:** Y. Z. Xie, R. X. Zhang

**Affiliations:** Department of Neurology, The Third Xiangya Hospital, Central South University, Changsha, 410013 Hunan Province China

**Keywords:** Induced pluripotent stem cells, IPSCs, Neurodegenerative disease, Disease modeling, Drug screening, Cell therapy

## Abstract

The study of stem-cell biology has been a flourishing research area because of its multi-differentiation potential. The emergence of induced pluripotent stem cells (iPSCs) open up the possibility of addressing obstructs, such as the limited cell source, inherent complexity of the human brain, and ethical constrains. Though still at its infancy phase, reprogramming of somatic cells has been demonstrating the ability to enhance in vitro study of neurodegenerative diseases and potential treatment. However, iPSCs would not thoroughly translate to the clinic before limitations are addressed. In this review, by summarizing the recent development of iPSC-based models, we will discuss the feasibility of iPSC technology on relevant diseases depth and illustrate how this new tool applies to drug screening and celluar therapy.

## Introduction

Neurodegenerative diseases occur as a result of neurodegenerative processes, including chronic and progressive loss of structure or function of neurons. Compared with other organs, recent progress in the molecular basis of neurodegenerative disorders has evolved slowly. This mainly due to brain tissue samples were rarely available for obtaining and often demonstrated the final phase of the diseases. These limitations impede our progress for better understanding of disease onset and mechanisms [1]. Although genetically engineered animal models have achieved promising advances, these systems are still inadequate and do not faithfully mirror the disease mechanism due to species differences and genetic backgrounds. Possessing the developmental potential to form trophoblast and differentiate into three embryonic germ layers, human embryonic stem cells (hESCs) could be a new paradigm [2]. However, the success rate of hESC establishment is extremely low, combining with lacking of oocyte donors and ethical issues further restricted the extensive application. The recent method of obtaining induced pluripotent stem cells (iPSCs) from somatic cells offer unprecedented and exciting opportunities to solve multiple drawbacks of various models mentioned above. The aim of this review is to assess the recent literatures and key findings on modeling neurodegenerative disease using iPSCs. The potential of iPSCs as an ideal platform for drug screening and cell therapy are presented. Besides, limitations and challenges in iPSC modeling will also be discussed.

## The developmental process of iPSC technology

Yamanaka first illustrated how cell fates rewound to a pre-embryonic state using retroviruses and lentiviruses by the ectopic co-expression of transcription factors [3, 4]. That discovery won him the Nobel Prize for Medicine in 2012. These iPS cells subsequently converted to specific cells of interest, such as neurons and glia that are relevant for different neurodegenerative diseases. However, it is also time-consuming, expensive. Moreover, this new approach possesses the possibility of oncogene reactivation when the cocktail of reprogramming factors contained proto oncogene *c*-*MYC* and the risk of insertional mutations. To reduce the potential adverse effects and to emphasize the need to safety, hiPSCs have been established by excluding *c*-*MYC* of four conventional reprogramming factors [5, 6]. Several groups took advantages of adenoviral and plasmid for non-integrating which avoid tumor formation, however, these manners are very labor intensive and the efficiency in general is extremely low [7, 8]. Sendai virus, protein, modified mRNA, and Micro-RNA have been used to generate iPSCs with no genomic integration [9–13]. The disadvantages of each vector type are still apparent. For example, the problems of purging cells of replicating virus and sequence-sensitive RNA replicase when applying Sendai virus can not be ignored [13]. Multiple rounds of transfection using modified mRNA vector were needed to achieve controllable and high-efficiency goal [12]. Recently, researchers have obtained mouse iPSCs from somatic cells using a combination of small-molecule compounds because they are cost-effective, easily synthesized, and nonimmunogenic [14]. Important progresses of iPSC-based scientific exploration have already been made on neurological diseases, haematological diseases, cardiac diseases, liver related diseases, etc. Here, we focus on current-established iPSCs models on neurodegenerative disorders (summarized in Table 1).

**Table 1:** Summary of reported iPSC-based disease models for neurodegenerative diseases

Disease	Associated gene	Primary cells	Target cell differentiation	Disease-specific phenotype	Therapeutic response	References
PD	*LRRK2*	Fibroblasts and epidermal keratinocytes	Dopaminergic neurons	Yes	Mutant allele correction	[17–19]
	*PINK1*	Fibroblasts	Dopaminergic neurons	Yes	PINK1 overexpression	[20]
	*PARK2*	Fibroblasts	Dopaminergic neurons	Yes	Lentiviral expression of Parkin	[21, 22]
	*SNCA*	Fibroblasts	Dopaminergic neurons	Yes	Mutant allele correction	[23, 24]
	*GBA1*	Fibroblasts	Dopaminergic neurons	Yes	Mutant allele correction	[25]
AD	*PS1* and *PS2*	Fibroblasts	Neurons	Yes	γ-Secretase inhibitors and modulators	[28, 29]
	*APP*	Fibroblasts	Neurons	Yes	β-Secretase inhibitors	[30]
	Sporadic	Fibroblasts	Neurons and astrocytes	Yes	Docosahexaenoic acid	[31]
HD	*HTT*	Fibroblasts	Neural stem cells, neuronal precursors striatal neurons, and astrocytes	Yes	Proteasome inhibitors and normal repeat substitution	[34–39]
ALS	*SOD1*	Fibroblasts	Motor neurons	Yes	Conditional expression of neurofilament-l	[43, 44]
	*TDP*-*43*	Fibroblasts	Motor neurons and astrocytes	Yes	Anacardic acid	[45, 46, 48]
	*VAPB*	Fibroblasts	Motor neurons	Yes	No	[47]
SMA	*SMN1*	GM09677 fibroblasts	Motor neurons	Yes	Valproic acid, tobramycin, SMN overexpressing and gene correction	[51–55]
FRDA	*FXN*	Fibroblasts	Peripheral sensory neurons and neural crest progenitors	Yes	No	[60, 61]
MJD	*ATXN3*	Fibroblasts	Neurons	Yes	Calpain inhibition	[63]
FD	*IKBKAP*	Fibroblasts	Neural crest precursors and neurons	Yes	Kinetin, glucosaminic acid, SKF-86466, phenindione, etc.	[65–67]

### Parkinson’s disease

Parkinson’s disease (PD) is the most common form of a group of progressive neurodegenerative movement disorders, and the prevalence is about 100–300 per 100,000 [15]. Initial PD-iPS cells were generated to reveal aspects of the abnormalities of PD, but no relevant phenotype was evident in the study [16]. Later, iPSC lines from a patient harboring a mutation in *LRRK2* gene were found partially consistent with early PD phenotype of enhanced susceptibility to oxidative stress (OS) [17]. In *LRRK2* G2019S iPSC-derived dopaminergic (DA) neurons, morphological alterations were observed when compared to control lines, with neurons showing decreased neurite length and reduced numbers of neuritis. This study also observed accumulation of autophagosomes and reduction of autophagic flux [18]. Recently, the phenotypes in two patient-derived iPSC lines with *LRRK2* G2019S mutation were rescued by specifically correcting the corresponding mutant allele. Moreover, certain experiment highlighted an elevated level of α-synuclein and *MAPT* expression in counterpart lines while not in the *LRRK2* genetically corrected lines [19]. Mutations in either *PINK1* or *PARK2* cause recessive forms of inherited PD characterized by impaired mitophagy because PINK1 and Parkin proteins function together. A study, including three patients with missense (c.509T>G; p.V170G) in mitochondrial protein *PINK1*, reported that the PINK1 mutant phenotype could be reversed after overexpression of wild-type *PINK1* [20]. In studies examining iPSC cells from patients with *PARK2* mutations, iPSC-derived neurons demonstrated functional and morphological abnormalities of mitochondria along with increased oxidative stress, rather than iPSCs or fibroblasts [21]. Similar to Imaizumi and colleagues, Jiang et al. [22] demonstrated altered features in mutant DA neurons, including enhanced sensibility to OS and monoamine oxidases activity, increased spontaneous DA release, and decreased DA uptake. These phenotypes were reversed through lentiviral expression of Parkin. Human iPSC-derived lines with mutations in *SNCA* expressed double the amount of α-synuclein when differentiated into DA neurons, while not seen in the original fibroblasts [23]. By use of zinc finger nuclease-mediated genome editing technology, researchers corrected the underlying point mutations (A53T) in *SNCA*-PD-iPSC. However, the phenotypes of iPSC-derived neurons in this work were not assessed [24]. A more recent study applied iPSCs possessing *GBA1* mutations to the modeling of PD. Schondorf et al. [25] observed increased levels of α-synuclein and glucosylceramide as well as lysosomal and autophagic defects in neurons from *GBA1* PD-iPSC. Importantly, these pathological phenotypes could be rescued by correction of the mutations.

### Alzheimer’s disease

Alzheimer’s disease (AD) is caused by progressive degeneration and loss of neurons and synapses throughout the brain [26]. Most of early-onset familial AD (FAD) can be attributed to mutations in one of three genes: *APP, PS1* and *PS2*, while many genetic and environmental factors may co-contribute to determining the sporadic AD (SAD) [27]. The investigation of AD was extremely limited until FAD-derived iPSCs with *PS1* (A246E) and *PS2* (N141I) mutations were established. In FAD-iPSC-derived differentiated neurons, the secretion of amyloid-beta (Aβ) was significantly increased and sharply responds to γ-secretase modulators and inhibitors [28]. Furthermore, iPSC-derived neuronal cells showed AD-like biochemical features, such as an increase in Aβ ratio. These neurons have reduced to secrete Aβ when treated with β-secretase inhibitor, γ-secretase inhibitor, and a nonsteroidal antiinflammatory drug. During the differentiation stages, however, there existed different susceptibilities to these drugs [29]. Other iPSCs model was established from FAD patients with a duplication of *APP* gene and two cases of SAD. Higher level of phospho-tau and glycogen synthase kinase-3 in lines with elevated Aβ represented novel observations in neurons from one of the SAD cases. Interestingly, these alterations could be alleviated by β-secretase, not γ-secretase, inhibitor treatment [30]. Docosahexaenoic acid (DHA) may actually be effective for some patients for the reason that the stress responses in the AD neural cells were alleviated when treated with DHA, making possible the iPSC technology for validation and identification of promising drugs [31].

### Huntington’s disease

Huntington’s disease (HD) is a progressive neurodegenerative genetic disease associated with a wide variety of motor impairment and psychiatric symptoms caused by excessive CAG trinucleotide expansions of *Huntingtin* gene (*HTT*) [32]. The first established human HD-iPSC model from HD patients carrying 72 CAG repeats was reported in 2008 [33]. Striatal neurons and HD neural stem cells produced from iPSCs exhibited elevated caspase activity after the withdrawal of growth factors which indicate apoptosis, but no overt cell death phenotype was reported [34]. Experimental analysis of proteins revealed amounts of distinct alternations in protein expression in the HD iPSCs [35]. Jeon et al. converted HD-iPSC into GABAergic striatal neurons and relavent behavior recovered in a grafted rat after transplantation of HD-iPSC derived neural precursors. Though showing no overt HD phenotype, the disease-specific iPSCs sharply responded to proteasome inhibition and expressed several markers of HD pathology after transplantation at later time points [36, 37]. Additional HD-phenotypes, including altered mitochondrial bioenergetics and susceptibility to cell death were reported in a subsequent study. These alterations accompanied with pathogenic HD signaling pathways could be reversed by the substitution of a normal repeat for the expanded CAG repeat using homologous recombination [38]. A more complete study by the HD Consortium demonstrated hundreds of distinct differences between HD and wide-type (WT) iPSCs, showing clear signs of HD-related pathology in several lineages. The lines with the longer CAG repeat expansions had more severe pathological phenotypes and ultimately lead to neuronal death [39]. These findings revealed that HD-iPSCs could be a well-characterized and unique resource to elucidate disease mechanisms.

### Amyotrophic lateral sclerosis

Amyotrophic lateral sclerosis (ALS) is the most common form of progressive motor neuron disorder with varied etiology characterized by rapidly progressive weakness and muscle atrophy. This disease strikes people in their 40s, approximately 20 % of familial ALS cases were linked to dominant mutations in the *SOD1* gene [40]. Other different genes, including *VAPB, TDP*-*43* and *FUS* have also been implicated in ALS [41, 42]. ALS-iPSCs were developed using skin fibroblasts from two octogenarian sisters with *SOD1* mutations and then converted into spinal motor neurons, but no assay of ALS relevant phenotype or compared with neurons from controls were performed in this study [43]. Another iPSC-derived motor neurons (MNs) possessing *SOD1* mutations developed neurofilament (NF) inclusions. Importantly, conditional expression of NF-L in these MNs corrected the NF subunit proportion, mitigating NF aggregation and neurite degeneration [44]. The mutant astrocytes from iPSCs harboring a mutation in the *TDP*-*43* gene exhibited subcellular mislocalization of TDP-43, increased levels of TDP-43, and reduced cell survival. Further co-culture assays showed these astrocytes did not adversely affect survival of cocultured neurons [45]. Another *TDP*-*43* iPSC-derived MNs recapitulated the disease phenotypes, including decreased survival, increased cellular vulnerability, and elevated levels of soluble and detergent-resistant TDP-43 protein [46]. Researchers also generated iPSCs from patients which carry the *VAPB* mutation as well as from their healthy siblings. The finding suggested that reduced levels of VAPB protein in MNs could be involved in the pathogenesis of ALS8 [47]. Test for chemical compounds on differentiated MNs showed that the abnormal ALS MNs phenotype could be rescued by anacardic acid, suggesting that special MNs may be a new approach for elucidating ALS disease mechanisms and for screening candidate drugs [48].

### Spinal muscular atrophy

Spinal muscular atrophy (SMA) is an autosomal recessive genetic disease characterized by the loss of the survival motor neuron (SMN) [49]. There are two SMN genes in humans, *SMN1* and *SMN2*, and the loss of the *SMN1* gene primarily caused SMA [50]. To model SMA, scientists generated iPSCs from a child carrying *SMN1* mutation. These cells manifested clear signs of motor neurons and differentiated neurons showed the reduction in cells size and number compared to controls at later stages, indicating they underwent substantial degeneration. Excitingly, the administration of valproic acid and tobramycin could reverse the endogenous SMN protein level in differentiated neurons and astrocytes [51]. Another established neuronal cultures rescued the phenotype of delayed neurite outgrowth and restored normal motoneuron differentiation by overexpressing SMN, accelerating the exploration of the underlying mechanisms of SMA pathogenesis [52]. MNs from two type I SMA subject-derived iPSCs showed elevated activation of caspase-8 and-3 and increased Fas ligand-mediated apoptosis. Moreover, distinct inhibitors of apoptotic pathways may reduce motor neuron cell death [53]. Another study has focused on genetic correction for autologous cell therapy. Uncorrected SMA-iPSCs derived motor neurons manifested disease-specific features. Delightfully, these phenotypes were ameliorated in genetically corrected controls, suggesting that genetically corrected special motor neurons could be a source for future therapeutic strategy [54]. Novel observations of reduced androgen receptor levels, reduced HDAC6, and repeat instability provided evidence that SMA-iPSC derived MNs could be new avenues for further investigation of the disease mechanism and development of effective therapy [55].

### Inherited ataxias

Inherited ataxias may show autosomal dominant, autosomal recessive modes of inheritance. Friedreich ataxia (FRDA) is an autosomal recessive ataxia, accounting for one-half of all hereditary ataxias. Spinocerebellar ataxias (SCAs) are genetically defined autosomal dominantly inherited disorders characterized by progressive lack of motor coordination.

FRDA is caused by large guanine-adenine-adenine (GAA) expansions in *FXN* gene on chromosome 9q13, leading to a transcriptional defect of FXN mRNA and frataxin [56, 57]. GAA·TTC triplet repeats in *FXN* intron 1 in FRDA iPSCs not only expanded at a higher rates but also exhibited repeat instability [58, 59]. The mismatch repair enzymes MSH2 were much highly expressed in iPSCs than fibroblasts and neuronal stem cells. Moreover, a specific pyrrole-imidazole polyamide which displaced MSH2 in FRDA iPSCs could partially impede repeat expansion [59]. Studies above demonstrated that GAA repeats instability might start early during ontogenesis and the highly active mismatch repair system is related to the GAA·TTC triplet repeats. Another iPSCs presented an instable repeats of GAA expansion, but was failed to find biochemical phenotypes [60]. Recently, a new development-based differentiation protocol was applied to differentiate FRDA and control iPSCs into peripheral sensory neurons and neural crest progenitors. Increased expression of frataxin during sensory specification for control cells was identified compared to FRDA peripheral sensory neurons. Whereas, a pronounced deficiency of frataxin was observed in FRDA iPSCs and neural crest cells, rather than controls [61].

SCA3, also known as Machado-Joseph disease, is the most common subtype of SCAs in China, which is caused by an abnormal CAG trinucleotide repeat expansion in *ATXN3* gene [62]. The formation of sodium dodecyl sulfate insoluble aggregates was observed in l-glutamate-induced excitation of iPSC-derived neurons and calpain inhibition could abolish these pathological changes. However, inclusion bodies and increased cell death were not observed, suggesting the excitation-induced protein aggregation is an early event [63].

### Familial dysautonomia

Familial dysautonomia (FD) is a debilitating neurodegenerative disease and the most common mutation is in a splice site of the *IKBKAP* gene involved in transcriptional elongation, resulting in reduced levels of IKAP protein [64]. Researchers differentiated patient-specific FD-iPSCs into peripheral neurons directly. Tissue-specific mis-splicing of *IKBKAP* in vitro and low level of normal *IKBKAP* transcript expressed in patient neural crest precursors were observed. The potency treatment of rescuing the mutant splice combines with improving neuronal differentiation and migration were also concluded [65, 66]. Later, 6,912 compounds were screened, while 8 hits were characterized that rescued expression of *IKBKAP*. SKF-86466, one of 8 hits, not only induced *IKBKAP* transcription but also rescued the expression of IKAP protein and the disease-specific loss of autonomic neuron sign [67]. Taken together, small molecule discovery in these newly disease models can gain novel insights into human disease pathogenesis and promising treatment.

## Potential and challenges

The promising iPSC technology has the potential to model and treat neurodegenerative diseases. Patient-derived iPSCs can be rewound to affected neuronal subtypes via in vitro differentiation or repaired iPS cells using gene targeting to repair disease-causing mutation. One notable potential is to apply these affected neuronal subtypes to develop promising drugs that are most suitable for the patient associated with their efficacy and toxicity profile. The generation of iPSCs permits the production of large scale of central nervous system cell with specific genotype, providing an unlimited amount of experimental materials. The other potential is to develop autologous-repaired iPS cells for cell therapy. When transplanting desired cells into patients, neural function could be restored and long-term immuno-suppression may not be necessary. Moreover, transplantation of glial cells, such as astrocytes that would restore the motor neurons potentially, can also used for neuroprotection.

However, it is not until this technology ready for clinical applications that its limitations were overcame. Firstly, initial method that introduction of factors by retrovirus or lentivirus might provide the possibility of oncogene reactivation [3, 4]. Apart from safety, the genetic and epigenetic status of clones might have varied after reprogramming. Although the non-integrating approaches significantly reduced the risk of tumorigenesis and epigenetic variation, researchers need to be cautious and select the stable lineages for differentiation studies. Epigenetic modifications could be significant in disease manifestation and the erasure of epigenetic marks during reprogramming is important [7–13]. Secondly, how to generate disease-relevant phenotype in vitro is also a challenge. The appearance of disease phenotype is the most important feature for disease modeling. Current iPSC-models were generated from patients containing mutations in known gene. However, many patients, such as PD and ALS, have an unidentified genetic component that is coupled with environmental factors. Importantly, the majority of neurodegenerative disorders are age-dependent and age is considered to be a risk factor that contributes to the disease development. As current culture system involved no disease-triggering factors on disease, the iPSCs might no longer reflect relevant pathogenesis. Moreover, long-term culture was poorly controlled and could be compromised by excessive cell death. Thirdly, protocols for differentiating iPSCs towards different subtypes of neurons cannot be ignored. Up till now, greater progress has been made in generating matured populations of distinct neurons and glia for the purpose of screening drugs and replacement therapy. However, these cells may not truly reflect the cellular responses to compounds that the body would have at a physiological level. Lastly, researchers still face the problems of low efficiency conversion and laborious to conduct. The average conversion efficiency of each methods mentioned above is less than 1 %, which further restrict the extensively use of the technology [1]. Efficiency needs to be improved if these protocols apply for high-throughout drug screening.

## Conclusions

Taken together, iPSC technology for modeling of neurodegenerative diseases has both benefits and limitations. Patient-derived cells open new avenues to investigate the neurodegenerative diseases development and remarkably easily could usher in new therapies and cloning techniques. Many hurdles must be overcome before new clinical therapies based on these cells. This will help ensure safety and eventually beneficial to human beings.
